# Syphilitic Aortic Aneurysm in the Third Millennium

**DOI:** 10.1055/s-0039-1683399

**Published:** 2019-04-01

**Authors:** Federico Del Re, Giosuè S. Falcetta, Stefano Pratali, Beatrice Belgio, Angela Pucci, Uberto Bortolotti

**Affiliations:** 1 Section of Cardiac Surgery, University Hospital, Pisa, Italy; 2 Section of Pathology, University Hospital, Pisa, Italy

**Keywords:** aortic aneurysm, aortic valve replacement, ascending aorta

## Abstract

A 69-year-old man presented with precordial pain and a dilated ascending aorta with the suspicion of an intramural hematoma. At emergency operation, the aorta appeared grossly thickened with diffuse intimal scarring. Retrospectively, the patient tested positive to serologic screening for syphilis with histologic findings also compatible with a syphilitic aortitis.

A 69-year-old man, previously asymptomatic and with an unremarkable clinical history, presented with dyspnea and precordial pain at our emergency unit. He denied any risky sexual behavior and did not show any signs or symptoms related to secondary syphilis. An angio computed tomographic (CT) scan showed a fusiform aneurysm of the ascending aorta with a maximum diameter of 65 mm, with a thickened wall raising the suspicion of an intramural hematoma ( Fig. 1A ). Other aortic diameters were as follows: aortic root 35 mm, aortic arch 32 mm, and abdominal aorta 25 mm. The patient was transferred to the operating room for emergency operation without specific laboratory tests and coronary angiography. Intraoperative transesophageal echocardiography confirmed the presence of a dilated aorta with a fusiform aneurysm and prevalent moderate aortic regurgitation. The right femoral artery was isolated and cannulated, and after a median sternotomy, the right atrium was cannulated for venous return. Cardiopulmonary bypass was instituted, and after aortic cross-clamping, the ascending aorta was opened and the heart arrested with cold blood cardioplegia into the coronary ostia. At gross inspection, the aortic wall appeared severely thickened, with intimal scarring, tree-bark appearance, and calcific spots but without signs of dissection ( Fig. 1B ). The aortic valve showed fibrotic cusps and diffuse calcific spots whereas the aortic sinuses appeared not dilated. The valve was replaced with a 27-mm porcine bioprosthesis mainly because the surgeon considered it not reparable and being worried about its possible early further deterioration; the ascending aorta was also replaced with a 34-mm vascular graft reinforcing both proximal and distal anastomoses with felt. The patient recovered uneventfully and was discharged on postoperative day 14 on a regimen of penicillin. Retrospectively he tested positive to screening for treponema pallidum particle agglutination (1:640) and specific immunoglobulin G, whereas human immunodeficiency virus (HIV) test was negative. Specific tests to rule out central nervous system involvement were not performed before discharge. At the last follow-up visit 18 months following surgery, the patient was asymptomatic.

**Fig. 1:**
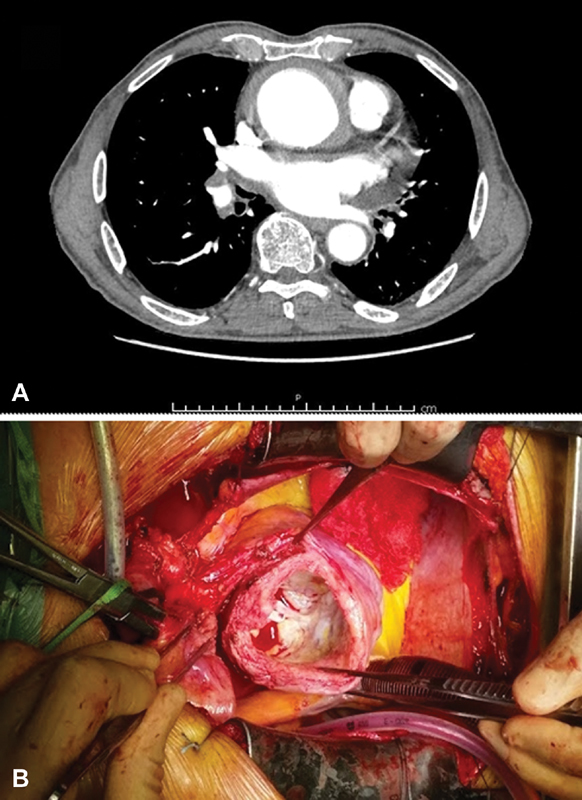
( **A** ) Marked dilatation of the ascending aorta on angio CT scan. ( **B** ) Intraoperative view showing a diffusely thickened aortic wall with *tree-bark* scarring.

Histology of the aortic wall showed full-thickness infiltrates involving all layers with adventitial fibrosis surrounding thickened vasa vasorum ( Fig. 2A , B ) with inflammatory infiltrates mainly constituted by CD138 positive plasma cells ( Fig. 2C ). Such findings coupled with laboratory data were considered compatible with a syphilitic aortitis.

**Fig. 2:**
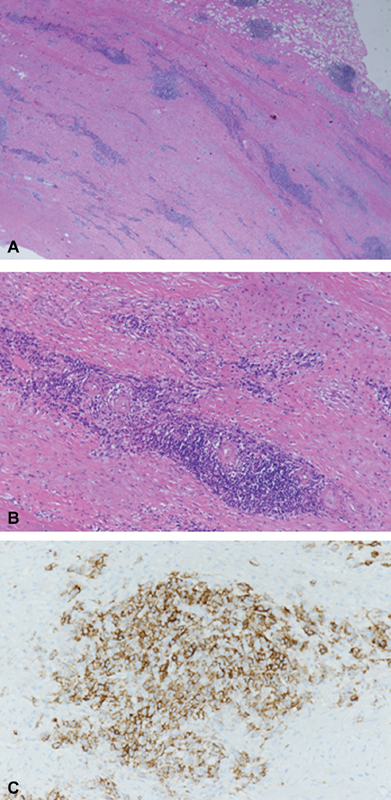
Histology of the aortic wall showing full-thickness infiltrates involving all layers with adventitial fibrosis ( **A** ) surrounding thickened vasa vasorum; ( **B** ) the inflammatory infiltrates are mainly constituted by CD138 positive plasma cells ( **C** ). (A, B) Hematoxylin-eosin staining, 2× (A) and 10× (B). ( **C** ) Immunoperoxidase technique with hematoxylin counterstaining, 20×.

Aortitis is the main manifestation of a syphilitic cardiovascular infection, but aortic aneurysms due to tertiary infection are substantially uncommon in the modern era; 1 recently, however, a review by Drago et al has found that approximately 71% of patients with syphilitic aortitis have an aortic aneurysm. 2 Liberal use of antibiotics has contributed to the considerable decline in late manifestations of syphilis. In fact, recent reports on this subject mainly consist of single cases of syphilitic aneurysms and their complications. 3 4 5

Syphilitic aortitis is usually diagnosed combining histologic and laboratory data, although cases of negative laboratory testing have been reported. 6 In our case, the specific etiology of the aortic disease was based on the histologic appearance of the aortic wall, but the gross appearance of a thickened wall with intimal scarring raised the suspicion of an aortitis. Indeed, such features have been related to endoarteritis of the vasa vasorum, which became occluded by lymphocytic infiltrates with ischemic weakening and subsequent degenerative changes of the aortic wall. 1

Our experience demonstrates that luetic infection still remains a potential cause of ascending aortic aneurysms. 1 Therefore, such patients should undergo strict surveillance following operation to detect possible further involvement of the distal aorta. 7 Awareness of the problem can provide a significantly effective treatment and adequate follow-up therapy for this condition.
